# Foaming Performance and FTIR Spectrometric Analysis of Foamed Bituminous Binders Intended for Surface Courses

**DOI:** 10.3390/ma14082055

**Published:** 2021-04-19

**Authors:** Krzysztof Maciejewski, Anna Chomicz-Kowalska

**Affiliations:** Department of Transportation Engineering, Faculty of Civil Engineering and Architecture, Kielce University of Technology, Al. Tysiąclecia Państwa Polskiego 7, 25-314 Kielce, Poland; akowalska@tu.kielce.pl

**Keywords:** paving grade bitumen, polymer modified bitumen, HiMA, mechanical water foaming, ATR-FTIR

## Abstract

This study explores the effects of foaming on three selected bituminous binders: 50/70 paving grade bitumen, 45/80-55 polymer modified bitumen and 45/80-80 HiMA binder. The first part of the investigations included the evaluation of the foaming performance in terms of foaming temperature and foaming water content with the utilization of desirability functions and based on the equality of maximum expansion ratio and bitumen foam half-life. The second part of the study investigated the effects of foaming on the chemical structure of the binders using Fourier-transform infrared spectroscopy. The results of the spectroscopic measurements permitted calculation of structural indices specific to functional groups associated with bitumen oxidation, as well as those indicative of elastomeric modification. The results have shown that the different types of bitumen exhibited different foaming characteristics, which was most evident in bitumen foam half-lives, with the HiMA binder performing the best. The spectrometric measurements did not show any significant effects of foaming on the chemical structure of the evaluated binders related to oxidative stress, neither were any major changes in the PmB-specific regions found.

## 1. Introduction

In the growth of environmental awareness and government-imposed regulations encouraging the shift towards a “green” economy, the road construction industry faces expectations for decreasing the energy-intensity of its operations while increasing the quality and longevity of the constructed infrastructure. These conditions require the introduction of new, energy efficient techniques (e.g., bitumen foaming [1,2] and fluxing [3]) and materials (e.g., industrial by-products [4] and reclaimed materials [5,6]), while preserving the high performance of the produced infrastructure. In this scope, the utilization of various foaming processes for producing bituminous mixtures for road courses is notably adequate [7,8]. Specifically, the “mechanical foaming” of bitumen, utilizing direct injection of water to hot bitumen, provides a straightforward and cost-efficient solution for decreasing the processing temperatures and energy requirements for producing bituminous mixtures [9,10,11,12]. Foamed bitumen is now widely used for producing high quality cold recycled mixtures [1,13,14,15,16] as well as high performing warm mixtures, enabling use of recycled materials and resembling typical hot mix asphalt in their properties [1,17,18,19,20].

The increased popularity of warm mix asphalt with foamed bitumen created scientific interest in the properties of foamed bitumen and long-term durability of foamed mixtures. The properties of mineral bitumen mixtures, among other factors, are highly dependent on the composition, temperature and aging history of bituminous binders [21,22,23]. Different approaches to evaluating the foamability of bitumens are known, some originating in the technology of cold mixtures [7,24], while others were investigated in the scope of the properties of warm mixtures [9,24]. The recent works of Bairgi et al. [25,26] have shown that the mechanical responses (stiffness, viscosity and others) of foamed bitumen change rapidly up to ca. 1.5% FWC, but a further increase in FWC does little change to those parameters. The works of Yin et al. [9] and Newcomb et al. [27] regarding the properties of WMAs with foamed bitumen also show that the best properties of these mixtures are achieved when low FWCs are used (typically <2%). These results show that obtaining high quality WMA mixtures with foamed bitumen is possible without conforming to the high foamability limits established for cold and half-warm mixtures.

In recent years, a number of studies have been published regarding the effects of foaming on the properties of bituminous binders. Bairgi et al. [26,28,29] and Maciejewski [21], in their independent studies, have not found clear evidence for detrimental effects of foaming on high temperature rheological and functional properties of bituminous binders. Sunarjono [30] has come to similar conclusions based on investigations of the consistency of foamed bituminous binders. Huang et al. [31], in their neutron scattering experiments, found no microstructural changes in bitumen subjected to foaming. Some other studies [32,33,34,35] on the other hand, have shown significant effects of foaming on the high- and low-temperature properties of paving grade and polymer modified bitumen.

On the side of water-bitumen interactions, Hung et al. [36] investigated the effects of water exposure at elevated temperatures on the surface microstructure and chemical composition using atomic force microscopy and infrared spectroscopy. In this study, significant topological and chemical changes on the surface of the investigated binders were found; all of which, however, were reversible after annealing of the binder. Huang and Turner [37] aged different bitumen in a humid environment at atmospheric pressure. Their results showed that there was no significant influence of moisture on aging kinetics, although it should be noted that the spectrometric measurements were taken using a solvent.

Similar conclusions can be drawn from the studies conducted during the construction of test sections using various warm-mix techniques, where the material produced using foaming techniques did not exhibit excessive aging in the early years of service [38].

Based on the presented state-of-the-art in the subject area, a study was conducted to first investigate the effects of foaming on three, distinctly different, bituminous binders widely used for producing surface courses: an ordinary paving grade bitumen and two elastomer-modified binders. The first part of the study was aimed at evaluating the foaming performance using different criteria, while the second part was to investigate whether water foaming induced changes in the chemistry of the bituminous binders, specifically those associated with oxidative stress.

## 2. Materials and Methods

### 2.1. Materials and Experimental Plan

The study investigated the effects of foaming on a road paving bitumen, polymer modified bitumen and a highly modified binder (HiMA), all representing grades typically used in surface course asphalt mixtures. The investigated commercial binders sourced from a local refinery (Orlen Asfalt, Płock, Poland) were denoted as PGB (50/70, Paving Grade Bitumen), PMB (45/80-55, Polymer Modified Bitumen) and HMB (45/80-80 HiMA, Highly Modified Bitumen). The basic properties of the bituminous binders are given in Table 1. The experimental plan included two successive stages of investigation, first, for evaluating foaming performance of the investigated binders and, second, for assessing the influence of foaming on their chemical composition.

**Figure 1:**
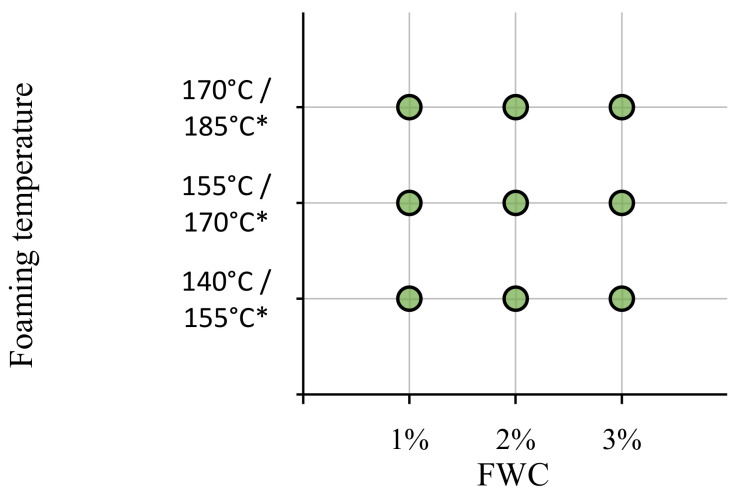
Experimental plan for foaming of investigated binders; asterisk (*) denotes temperature range for foaming of the HMB (45/80-80 HiMA) binder.

In the first stage, the bitumens were tested for their foaming performance. The tests were designed to evaluate the effects of temperature and foaming water content (FWC) on the maximum expansion (ER_m_) and half-life (T_1/2_) of the bitumen foam. Based on these results, an optimization analysis was conducted to establish such parameters of the foaming process which would result in an optimum foaming performance of these binders. Figure 1 presents the experimental plan for the evaluation of foaming performance of the PGB, PMB and HMB bituminous binders.

The experiments featured three factors evaluated on three levels (bitumen foaming temperature and foaming water content) with three replicates at each factor level. The foaming water content was controlled at 1%, 2% and 3% for all binders, but the foaming temperature ranges depended on the type of the binder. The foaming of the 50/70 and 45/80-55 binders was conducted at 140 °C, 155 °C and 170 °C, whereas the 45/80-80 HiMA bitumen was foamed at 155 °C, 170 °C and 185 °C. This distinction had multiple origins. The lowest values of foaming temperatures were mainly established based on the manufacturer’s recommended (Table 2, [39]) minimum pumping temperatures, which are a function of dynamic viscosities of the binders at high temperatures. Additionally, the authors’ previous experiences showed that a sufficiently high foaming temperature is needed to vaporize the foaming water and provide the effectiveness and sustainability of the foaming process. The highest values of the foaming temperatures were defined by the binders’ resistance to high temperatures as given by the manufacturer (Table 2, [39]). Finally, the experiments were set up with equal temperature steps.

In the second part of the study, the three binders were evaluated before and after foaming at the selected optimal process parameters (temperature, FWC). Here, the formation of oxidative ageing products and the chemistry of elastomeric modification were investigated using FTIR spectrometry with three replicates.

### 2.2. Methods

#### 2.2.1. Measuring and Evaluating Bitumen Foaming Characteristics (ER_m_ and T_1/2_)

Similar to other works (e.g., [27,40]), the foaming trials were conducted with the use of a Wirtgen WLB 10 laboratory foamer (Windhagen, Germany) and the foaming performance was evaluated based on the following properties of the bitumen foam, acquired using a steel measure and a stopwatch [7,11,27]:

- Maximum expansion ratio (ER_m_)—defined as the maximum increase of the bitumen volume due to foaming;
- Half-life (T_1/2_)—defined as the amount of time passed between the measurement of maximum expansion ratio and the halving of the bitumen foam volume.

The foaming conditions (air and water pressure) were in accordance with [41,42]. All test samples were prepared according to EN 12594:2014-12.

The foaming results were subjected to statistical analysis in order to evaluate the effects of the controlled variables on the expansion ratio and half-life characteristics. To facilitate this, the experimental data were fitted using second order linear statistical models with interaction terms and adequate analyses of variance were computed [43]. To visualize the gathered data, corresponding response surfaces were graphed, showing the relationships between foaming temperature, foaming water content and the ER_m_ and T_1/2_ for each individual bitumen.

#### 2.2.2. Simultaneous Optimization of Foaming Performance

The formulated statistical models describing the relationships between the foaming characteristics and the foaming process parameters were used to establish optimal values of foaming temperature and foaming water content for each bitumen. Two different approaches were taken in order to accomplish this aim.

**Figure 2:**
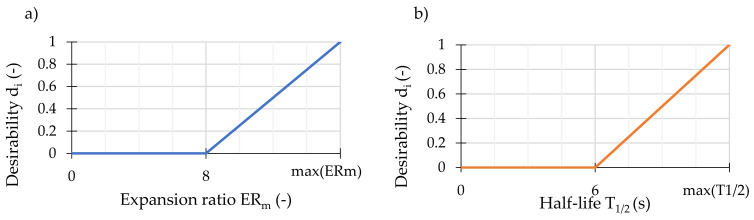
The desirability functions used for converting: (**a**) the predicted expansion ratios and (**b**) bitumen foam half-lives into desirability values.

The first method based on the principles of simultaneous optimization of multiple variables, utilized desirability functions for computing desirability indices, described in detail in [44,45,46,47]). This method permitted the identification of bitumen temperatures and foaming water contents at which the expansion ratios and bitumen foam half-lives simultaneously reached the highest values. The formulation of the desirability functions used in this study is shown in Figure 2. The presented criteria were adopted after [48], as they are relatively liberal among those proposed in the literature [7,48,49,50]. To this date, there are no unequivocal recommendations on bitumen foamability for warm mixes [27,40]. The desirability indices were calculated as a geometric mean of the values of desirability functions as in [47].

The second method to optimize the foaming performance depended on finding the parameters of the foaming process that resulted in obtaining numeral equality of the ER_m_ and T_1/2_ values, interpreted as their balance [51]. This aim was achieved by calculating the value of logarithmic Equation (1), based on the predicted values of expansion ratios and half-lives computed in small intervals in the whole domain of the experiment.

1 $$\log_{10}({\mathrm{ER}}_{m}/T_{1/2})$$

When the two evaluated values were equal, by the definition of the logarithm, the Equation (1) returns 0. Additionally, this expression computes symmetrical (in respect to 0) values for the opposite ER_m_ and T_1/2_ ratios, e.g., “1” for 10:1 ratio and “−1” for 1:10 ratio. This fact makes it effective for comprehensible graphing of the values of the ${\mathrm{ER}}_{m}/T_{1/2}$ ratio.

#### 2.2.3. Spectrometric Analysis

The methods for the identification and quantification of chemical compounds using infrared spectroscopy emerge from the principles of quantum mechanics and the structure of the individual compounds. Chemical molecules are built of atoms of a certain mass. These are connected by elastic bonds, which permit specific vibrations depending on the masses of the atoms, their geometrical arrangement and the strength of those bonds. A molecule that is irradiated with a spectrum of infrared radiation takes up a specific amount of the energy which excites a specific vibrational state, leaving out a remaining radiation spectrum with an absorption band at a frequency corresponding to the mode of vibration. Based on those principles it is possible to identify and quantify certain chemical groups and structures in bituminous binders, by calculating adequate indices [52,53].

In this study, attenuated total reflectance Fourier-transform infrared (ATR-FTIR) spectroscopy was used to evaluate the composition of the foamed binders. The Thermo-Scientific Nicolet iS 5 FTIR Spectrometer (Waltham, MA, USA) and the PIKE Technologies GladiATR (Madison, WI, USA) accessory with a diamond window was used. The bitumen samples after foaming were poured into 1 dm^3^ glass containers, reheated at 140 °C for 2 h and homogenized for uniform distribution of water during first stages of cooling at room temperature. The testing was conducted 3 days after foaming. Samples were transferred onto the ATR crystal mechanically, without reheating. Three different samples of each binder were tested and the potential effects of foaming on the binders’ chemical composition, specifically due to oxidative ageing, were measured by evaluating the formation of sulfoxide and carbonyl compounds [54]. Additionally, the presence of elastomeric additives was investigated in their specific absorption bands.

The quantitative evaluation of the mentioned compounds was conducted by calculating normalized indices as given in Table 3. The areas under the respective peaks were computed by the common tangent baseline method as proposed in [52].

## 3. Results and Discussion

### 3.1. Evaluation of Bitumen Foaming Performance

**Figure 3:**
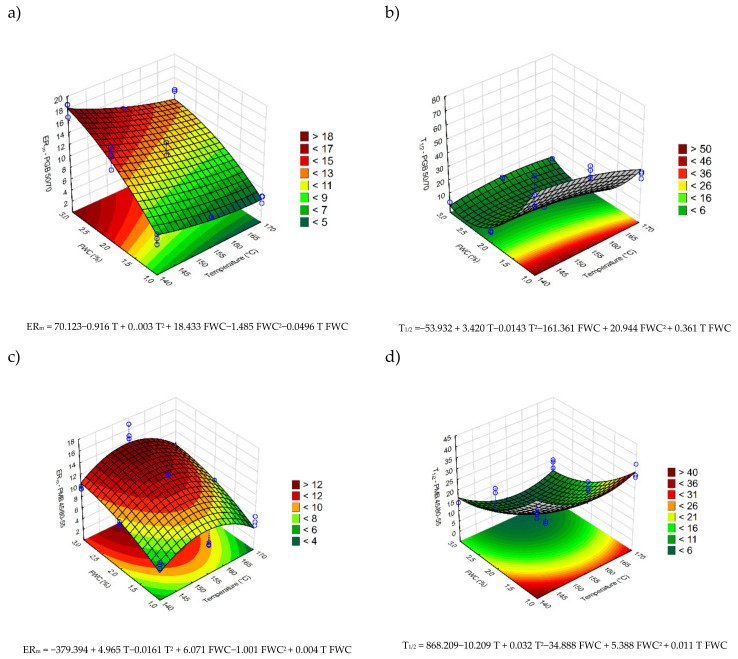

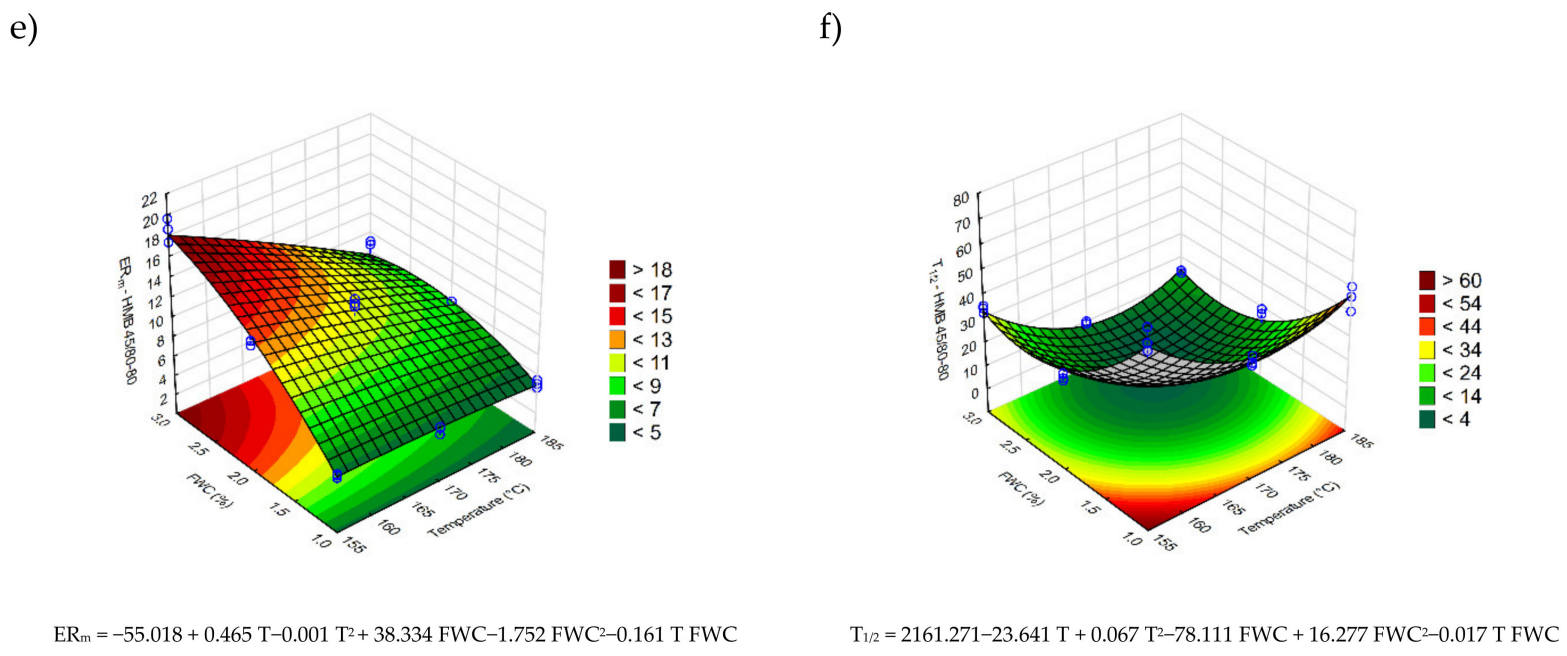
Response surfaces obtained through the measurements of ER_m_ and T_1/2_ foaming characteristics of: (**a**,**b**) 50/70, (**c**,**d**) 45/80-55 and (**e**,**f**) 45/80-80 binders.

The results of the evaluation of foaming performance of the discussed bituminous binders are presented in Figure 3. The graphs represent the response surfaces plotted based on the statistical models, which were fitted to the test data.

The statistical analysis presented in Table 4 shows that in all tested bituminous binders the expansion ratios and bitumen foam half-lives were significantly influenced by both the bitumen temperature and foaming water content. In most cases, those relationships were nonlinear, which was proven by the statistical significance of the quadratic and interaction terms.

In the case of all investigated binders, the increase in foaming water content resulted in increased maximum expansion ratio and decreased half-lives. The changes of expansion ratio due to varying FWCs were in most cases gradual and at least partly proportional to the amount of added water. The bitumen foam half-lives, on the other hand, were found to be rapidly decreasing when the FWCs changed from 1% to 2% and remained quite low at 3% FWC in the case of all binders.

The effects of temperature were more complex and depended on the type of the bitumen. In the case of the PGM 50/70 and HMB 45/80-80 binder the increase in temperature resulted in a steady increase in expansion ratio, whereas in the PMB 45/80-55 bitumen, a clear maximum of ER_m_ was observed at 155 °C. The bitumen foam half-lives of the 50/70 binder were, in practical terms, almost not influenced by the temperature of the binder. On the contrary, both polymer modified bitumen have shown significant minima of T_1/2_ in the middle of the evaluated temperature ranges.

**Figure 4:**
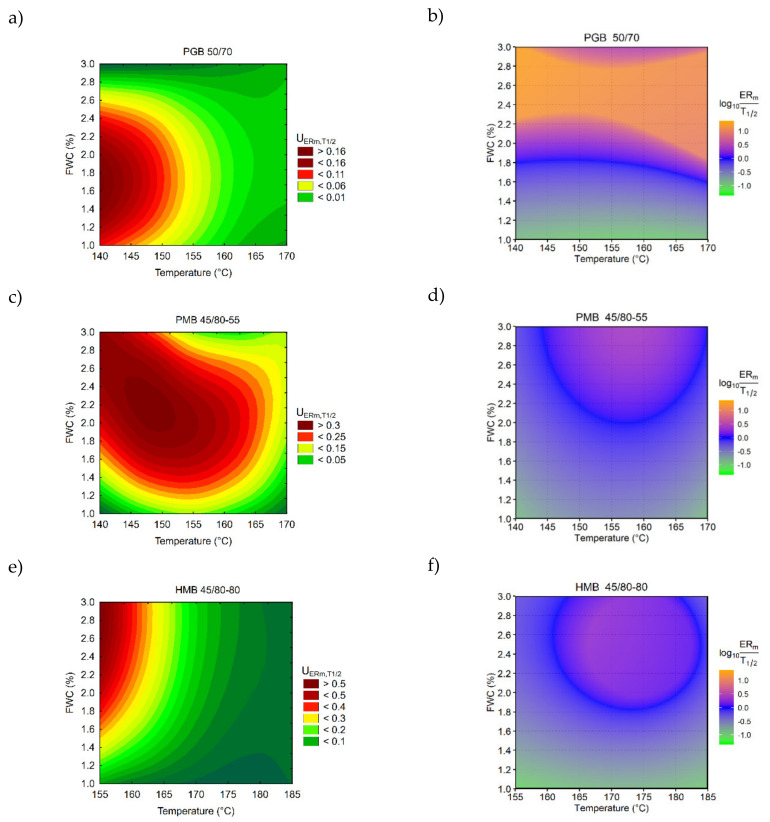
Optimization of foaming performance using: (**a**,**c**,**e**) desirability functions and (**b**,**d**,**f**) the equality of ER_m_ and T_1/2_ characteristics (values of “0” indicate the equality of ER_m_ and T_1/2_, i.e., ER_m_ = T_1/2_).

The results of the additional analyses for evaluating the foaming performance are presented in Figure 4. Regarding the 50/70 paving grade bitumen it was found that the highest ER_m_ and T_1/2_ were simultaneous achievable at lower temperatures (<155 °C) and in the range of 1–2% of foaming water content. At the same time, the equality of the foaming characteristics was found in the 1.6–1.8% FWC range throughout the temperature range. The 45/80-55 bitumen has exhibited the highest foamability in the middle of the temperature and foaming water ranges (Figure 4c), which at T = 155 °C and FWC = 2% also resulted in the balance of ER_m_ and T_1/2_ values. As for the HMB 45/80-80 binder, the analysis has shown that highest foamability was achieved at the lowest foaming temperature of 155 °C and high FWCs in the range of 2–3%. This effect was due to exceptional T_1/2_ values in the whole experimental domain and simultaneously high sensitivity of ER_m_ to changes in temperature and foaming water contents. The obtained equality region of ER_m_ and T_1/2_ in the HiMA binder was similar to that recorded for the PMB binder.

A significant observation was made during the experiments regarding the foaming of the HMB 45/80-80 HiMA binder. Although the foaming performance of HMB was assessed to be the best the lowest temperature of 155 °C, a high very resistance to flow was recorded, resulting in high pumping pressure and excessive strain on the bitumen pump.

In summary of the foaming performance evaluation, it can be stated that the tested binders showed different foaming characteristics; however, it was common for all the binders to exhibit equality of ER_m_ and T_1/2_ in the middle of the investigated temperature and foaming water content ranges. Based on these observations, the effects of foaming on the chemical composition of the bitumens were evaluated using binders foamed at 155 °C (50/70 PGB, 45/80-55 PMB) and 170 °C (45/80-55 HMB), in all cases utilizing 2% foaming water content.

### 3.2. Effects of Foaming on the Chemical Composition of the Binders

**Figure 5:**
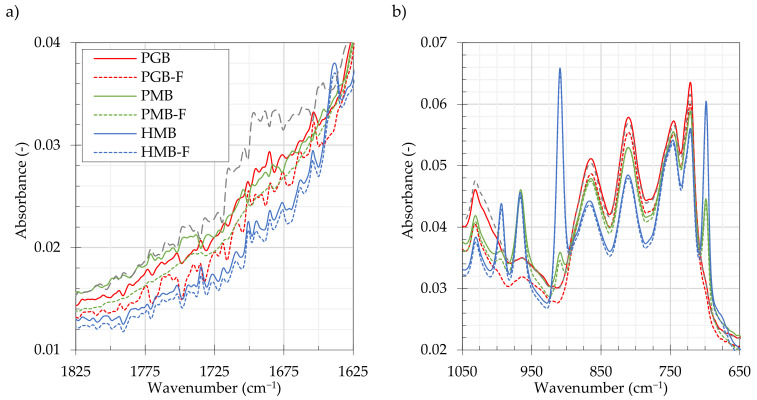
Infrared (ATR-FTIR) absorbance spectra of the evaluated binders before and after foaming in the 1625–1825 cm^−1^ (**a**) and the 650–1050 cm^−1^ (**b**) wavenumber ranges.

Figure 5 presents sample ATR-FTIR absorbance spectra of the tested PGB, PMB and HMB bitumen before and after foaming (annotated by “-F”). These spectra are supplemented by the results obtained by probing the PGB binder after RTFO aging (annotated by “-RTFOT”) to compare the effects of controlled oxidative stress induced by short-term ageing. Figure 5a shows the 1700 cm^−1^ wavenumber region, associated with the formation of carbonyl structures during oxidative aging. Figure 5b depicts the sulfoxide region (1030 cm^−1^) together with the peaks indicative of SBS polymer presence in the bitumen.

Figure 5a shows several distinct bands present in the 1650–1820 cm^−1^ carbonyl area, which could be attributed to specific groups of compounds in the carbonyl group. The bands between 1725–1775 cm^−1^ are characteristic of anhydrides, 1735–1750 cm^−1^ to esters, 1700–1714 to carboxylic acids, 1660–1700 cm^−1^ to ketones and 1640–1660 cm^−1^ to amides [53,61]. Figure 4a shows the changes in the absorbance spectra caused by the bitumen foaming as appearing relatively small. The additionally presented spectrum of the PGB-RTFOT sample subjected to a controlled oxidative stress presents significantly greater changes in the carbonyl region. The oxidation induced changes in the RTFO aged bitumen were evident in the wavenumbers between 1650–1715 cm^−1^, which, mostly being attributed to the formation of ketones, is in line with the conclusions of Petersen [54].

Figure 5b presents the 650–1050 cm^−1^ region of the ATR-FTIR absorbance spectra obtained for the evaluated binders. The well-pronounced peaks at 1030 cm^−1^ are indicators of sulfoxide presence which, next to carbonyl content, is one of the strongest predictors for rheological characteristics of bitumen [60]. This region also encompasses specific absorbance peaks, characteristic of SBS modified bitumen. The features recorded at 966 cm^−1^ and 699 cm^−1^ wavenumbers in the PMB and HMB binders can be related to the presence of polybutadiene and polystyrene, indicating a modification utilizing a styrene-butadiene-styrene (SBS) copolymer [57]. In addition to this, the HMB bitumen exhibited significant features at 910 cm^−1^ and 990 cm^−1^ wavenumbers, which could be related to the utilization of high vinyl SBS often seen in HiMA formulations [62,63]. The PMB and HMB binders after foaming showed negligible changes in the polybutadiene, polystyrene and vinyl specific regions of the spectra.

**Figure 6:**
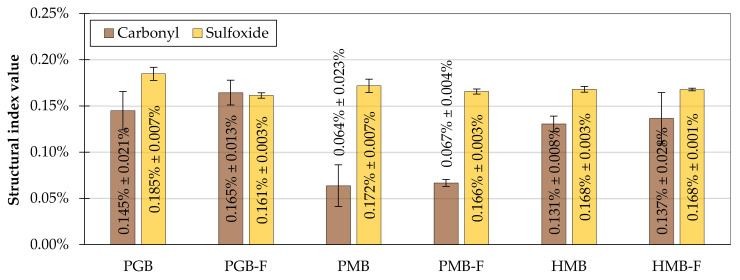
Carbonyl and sulfoxide structural indices calculated based on the ATR-FTIR measurements of the evaluated binders before and after foaming; error bars represent ±1 standard deviation from the mean.

Specific structural indices (Table 3) were calculated based on the FTIR measurements to objectively evaluate the effects of bitumen foaming on their chemical structure. The carbonyl and sulfoxide indices were used to evaluate whether this process induced oxidative ageing in the investigated binders. The values of the mean calculated carbonyl and sulfoxide indices are presented in Figure 6. Adequate statistical evaluation using two-way analysis of variance for evaluating the effects of bitumen type and foaming is shown in Table 5.

In the case of all investigated binders, the changes in mean values of the carbonyl and sulfoxide indices induced by bitumen foaming were small, both in absolute terms as well as in relation with the recorded standard deviations. The carbonyl indices tended to increase after foaming, whereas the sulfoxide indices decreased slightly. The greatest differences in the measured indices were observed in the PGM binder. The statistical evaluation (Table 5) has shown that only the change of the sulfoxide index in the PGM binder (its decrease) was statistically significant.

**Figure 7:**
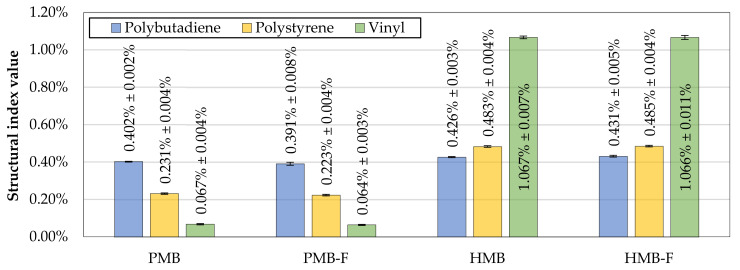
Polymer modification-specific structural indices calculated based on the ATR-FTIR measurements of the evaluated binders before and after foaming; error bars represent ±1 standard deviation from the mean.

Figure 7 presents the values of structural indices specific to elastomeric modifications with SBS copolymers, measured before and after foaming of the PMB and HMB binders. Table 6 presents a corresponding statistical evaluation of the effects of bitumen type and foaming on the values of those indices using two-way analysis of variance.

The values of the measured polybutadiene and polystyrene indices in the PMB binder indicated a typical linear SBS copolymer modification [63,64], while the values of vinyl indices in the HMB binder were characteristic of high vinyl SBS. The relatively high values of polybutadiene and polystyrene indices together with a very high vinyl index may be related to overall high SBS content, which is expected in a HiMA binder.

The measured structural indices related to the SBS modification remained virtually unchanged by the foaming process and the calculated variability of the measurements was very low. The results of the statistical evaluation showed no evidence that foaming had significant effects on the values of PmB specific structural indices.

## 4. Conclusions

The presented paper explored the effects of foaming on three distinctly different bituminous binders: 50/70 paving grade bitumen, 45/80-55 polymer modified bitumen and 45/80-80 HiMA binder. The significance of this work relates to the high requirements regarding bitumen used for surface courses due to their direct exposure to such factors as traffic loads, abrasion, as well as water and moisture.

In terms of the foaming performance, it was found that high values of expansion ratio and bitumen foam half-life, as well as the equality of those characteristics, could be simultaneously produced at intermediate foaming water contents, that is, FWC = 1.8–2.0%. Additionally, the HiMA binder exhibited the highest bitumen foam half-lives, while all bitumen were able to produce similar maximum expansion ratios under certain foaming conditions.

The FTIR analysis showed that no significant oxidative stress was induced due to mechanical water foaming of the tested bituminous binders. The tested polymer (SBS and high vinyl SBS) modified binders have also not shown any significant changes in the PmB-specific regions.

By the analysis of the presented results and the subject literature, it seems that the measured effects of foaming depend heavily on the type of measurement, sample conditioning as well as sample handling and preparation procedures. The available body of research suggests that the changes that foamed bitumen binders undergo, and which permit the decrease in processing temperatures of foamed bitumen mixtures are reversible during pavement construction and its early service life.

## Figures and Tables

**Table 1:** Properties of the investigated binders.

Binder Type		PGM 50/70	PMB 45/80-55	HMB 45/80-80
Penetration, EN 1426, (0.1 mm)		55	60	62
Softening point, EN 1427, (°C)		48.8	58.3	95.1
Dynamic viscosity, EN 13302, (Pa·s)	135 °C 155 °C 185 °C	0.48 0.22 -	1.14 0.39 -	2.91 0.65 0.19
Performance grade, AASHTO M 332		64–22	70–22	76–28
MSCR at 60 °C, EN 16659 (after RTFOT)	J_nr 3.2 kPa_ (kPa^−1^) R_3.2 kPa_ (%)	1.47 -	0.31 58	0.03 95

**Table 2:** Boundary processing temperatures of the investigated binders [39].

Binder Type	PGM 50/70	PMB 45/80-55	HMB 45/80-80
Min. pumping temp.	130 °C	150 °C	160 °C
Max. short-term storage temp.	185 °C	180 °C	180 °C

**Table 3:** Structural indices calculated for the bituminous binders [55,56,57,58,59,60].

Structural Index	Bond	Characteristic Peak WaveNumber (cm^−1^)	Chemical Index Expression:
Sulfoxide	S=O, stretching	1030	$I_{S=O}=\frac{A_{1030}}{\Sigma A_{\mathrm{all}}}$
Carbonyl	C=O, stretching	1700	$I_{C=O}=\frac{A_{1700}}{\Sigma A_{\mathrm{all}}}$
Polybutadiene	C–H, oop bending of trans-alkene	966	$I_{\mathrm{PB}}=\frac{A_{966}}{\Sigma A_{\mathrm{all}}}$
Polystyrene	C–H, oop bending in monoakrylated aromatic	699	$I_{\mathrm{PS}}=\frac{A_{699}}{\Sigma A_{\mathrm{all}}}$
Vinyl	=C–H oop bending in vinyl groups	990, 910	$I_{\mathrm{Vi}}=\frac{A_{910}+A_{990}}{\Sigma A_{\mathrm{all}}}$
*ΣA_all_ = A_(2953, 2923, 2862)_ +A_1700_ + A_1600_ + A_1460_ + A_1376_ + A_1310_+ A_1030_ + A_990_ + A_966_ +A_910_ + A_864_ + A_814_ + A_743_ + A_724_ + A_699_* oop—out of plane			

**Table 4:** Compound results of analysis of variance performed for assessing the significance of temperature and foaming water content on the foaming characteristics of the evaluated binders.

Binder Type	PGB 50/70		PMB 45/80-55		HMB 45/80-80	
Factor	*p*-Value (ER_m_)	*p*-Value (T_1/2_)	*p*-Value (ER_m_)	*p*-Value (T_1/2_)	*p*-Value (ER_m_)	*p*-Value (T_1/2_)
(1) Temperature (L)	<0.001	<0.001	0.005	<0.001	<0.001	<0.001
Temperature (Q)	0.194	0.058	<0.001	<0.001	0.428	<0.001
(2) FWC (L)	<0.001	<0.001	<0.001	<0.001	<0.001	<0.001
FWC (Q)	0.005	<0.001	0.001	<0.001	<0.001	<0.001
1L by 2L	0.038	<0.001	0.698	0.736	<0.001	0.750
Lack of fit	0.089	0.011	<0.001	<0.001	0.001	0.006
R^2^_adj_	0.924	0.949	0.763	0.768	0.951	0.966

**Table 5:** Two-way ANOVA statistical evaluation of carbonyl and sulfoxide structural indices in neat and foamed bituminous binders.

Effect	I_C=C_		I_S=O_	
	F	*p*	F	*p*
Intercept	760.07	<0.001	23849.35	<0.001
Bitumen	39.82	<0.001	2.12	0.163
Foaming	1.25	0.285	20.30	<0.001
Bitumen · Foaming	0.35	0.707	9.99	0.003

**Table 6:** Two-way ANOVA statistical evaluation of polymer modification-specific structural indices in neat and foamed bituminous binders.

Effect	I_PB_		I_PS_		I_Vi_	
	F	*p*	F	*p*	F	*p*
Intercept	81098.17	<0.001	91796.18	<0.001	81660.32	<0.001
Bitumen	124.16	<0.001	11996.03	<0.001	63870.71	<0.001
Foaming	1.20	0.304	1.43	0.265	0.31	0.595
Bitumen · Foaming	7.85	0.023	4.53	0.065	0.15	0.709

## Data Availability

Data available on request from the corresponding author.
